# Notes and News

**Published:** 1901-05-25

**Authors:** 


### Notes and News.

### HOSPITAL MEETINGS AND FESTIVALS.

King's College Hospital.—Annual Festival Dinner, Wednesday, June 12, at the Clothworkers' Hall, Mincing Lane, E.C., at 7 p.m., His Grace the Lord Archbishop of Canterbury presiding.

Westminster Hospital.—Festival Dinner, Wednesday, June 12, at Salters' Hall, St. Swithin's Lane, His Grace the Duke of Buccleuch presiding.

Royal Free Hospital.—Festival Dinner, July 15, at the Hotel Cecil, Right Hon. the Earl of Dartmouth presiding.

The Rev. S. Martyn Bardsley, M.A., the new Vicar of Greenwich, has been appointed Chaplain to the *Dreadnought* Hospital, Greenwich, with the understanding that the curates of the Parish Church, or some of them, will take part in the discharge of the chaplain's duties under his direction.

The officers, non-commissioned officers and men of the London Volunteer Medical Staff Corps gave a dinner at the Holborn Restaurant, last Saturday, to their comrades who had served in the Imperial Yeomanry hospitals in South Africa, and who recently returned to this country on the termination of their contracts.

A morning concert in aid of the East London Hospital for Children will take place in the Albert Hall on June 8th. Judging by the artistes who have promised to assist, and by the long list of lady patrons headed by H.R.H. Princess Christian, the concert ought to be a success, and we hope it will bring in a handsome addition to the income of this very deserving charity.

Lucky Scotland! Two millions to free the universities to her sons! When will London rear a Carnegie with such attachment to his native soil as to free or even to establish a university for Londoners. London, with cockney self-conceit regarding itself as the centre of the whole world, established a university, not for London alone but for the Empire—and beyond—and a very nice university, too, so far as giving degrees is concerned. Now that it has at last been discovered that degrees do not cover everything, the University of London has decided to teach as well as examine. For teaching, however, it is still in poor plight, and we have the sorry spectacle of the one sole university of the greatest city in the world not being able to pay for the class-rooms or the lecturers required to set it on its feet as a teaching institution. Lucky Scotland!

At the meeting of the Metropolitan Asylums Board held last Saturday Mr. R. M. Hensley was unanimously elected chairman of the Board for the year ending May, 1902, in succession to Sir E. Galsworthy, who has resigned. At the same meeting Dr. G. E. Shuttleworth, a medical examiner of defective children under the London School Board, was appointed for one year as medical expert in connection with Rochester House at a fee of five guineas for each day on which he may make visits in his official capacity, and Dr. R. H. Dixon was appointed for one year as visiting medical attendant at Rochester House upon the terms agreed to by the managers on October 20th last—viz., £100 per annum.

From the following summary supplied a few days since by the Countess Howe to the Central British Red Cross Committee, it will be seen that the Imperial Yeomanry Hospitals' Committee have sent out and maintained at the seat of war five distinct hospital sections, as well as a Convalescent Home at Johannesburg. The total subscriptions collected by the Committee amount to upwards of £145,000, by means of which the following staff and equipment have been sent to the seat of war:—Medical officers 50, dressers 24, nurses (Army Nursing Reserve Sisters) 102, ward maids 19, orderlies 495. 1,738 beds were provided, and the total number of patients treated to this date is 17,070, of whom 2,692 were treated on the field of battle by the Field and Bearer Company, in addition to 1,100 out-patients, including 265 Boer women and children.

We regret to announce the death of Dr. William Johnstone Fyffe, which took place on Friday at his residence, 2 Rodney Place, Clifton, Bristol, at the advanced age of 75. In 1854 he accompanied the 30th Regiment to Bulgaria, and subsequently joined the expeditionary force under Lord Raglan's command in their invasion of the Crimea. He was present at the battle of Alma. On his return to England he became one of the staff of the General Military Hospital, Fort Pitt, Chatham, where nearly all the wounded from the Crimea were collected. In 1863 he was appointed assistant professor of medicine in the Army Medical School at the Royal Victoria Hospital, Netley. Having completed 25 years' service in the Army, Dr. Fyffe retired in 1874 with the rank of deputy-surgeon-general. He was physician to Clifton College for a number of years.

Mr. H. C. Richards has given notice of his intention to move the rejection of the Bill enabling the Governors of Christ's Hospital to sell the Newgate Street site in order that it may be cleared and covered with commercial buildings. The contention of the Member for East Finsbury is that Parliament ought not to sanction the sale of any part of Christ's Hospital until a considered scheme for dealing with the land has been submitted to the authorities; such scheme to have regard alike to the reasonable requirement of the charity and of St. Bartholomew's Hospital, and to the importance of preserving some at least of the more interesting buildings of the hospital, and those portions of the site which have been used or set apart for interments, and which have hitherto existed as open spaces for the benefit of London, protected by the public Acts which prohibit building on disused burial grounds.

